# Increased Fear Memory and Glutamatergic Modulation in Compulsive Drinker Rats Selected by Schedule-Induced Polydipsia

**DOI:** 10.3389/fnbeh.2019.00100

**Published:** 2019-05-07

**Authors:** Ángeles Prados-Pardo, Elena Martín-González, Santiago Mora, Ana Merchán, Pilar Flores, Margarita Moreno

**Affiliations:** Department of Psychology, Health Research Center, University of Almería, Campus de Excelencia Internacional Agroalimentario CeiA3, Almería, Spain

**Keywords:** compulsivity, schedule-induced polydipsia, marble burying test, forced swimming test, elevated plus maze test, fear conditioning, glutamatergic modulators

## Abstract

Compulsive behavior is observed in several neuropsychiatric disorders such as obsessive-compulsive disorder (OCD), anxiety, depression, phobia, and schizophrenia. Thus, compulsivity has been proposed as a transdiagnostic symptom with a highly variable pharmacological treatment. Recent evidence shows that glutamate pharmacotherapy may be of benefit in impaired inhibitory control. The purpose of the present study was: first, to test the comorbidity between compulsivity and other neuropsychiatric symptoms on different preclinical behavioral models; second, to assess the therapeutic potential of different glutamate modulators in a preclinical model of compulsivity. Long Evans rats were selected as either high (HD) or low (LD) drinkers corresponding with their water intake in schedule-induced polydipsia (SIP). We assessed compulsivity in LD and HD rats by marble burying test (MBT), depression by forced swimming test (FST), anxiety by elevated plus maze (EPM) and fear behavior by fear conditioning (FC) test. After that, we measured the effects of acute administration (i.p.) of glutamatergic drugs: N-Acetylcysteine (NAC; 25, 50, 100 and 200 mg/kg), memantine (3.1 and 6.2 mg/kg) and lamotrigine (15 and 30 mg/kg) on compulsive drinking on SIP. The results obtained showed a relation between high compulsive drinking on SIP and a higher number of marbles partially buried in MBT, as well as a higher percentage of freezing on the retrieval day of FC test. We did not detect any significant differences between LD and HD rats in FST, nor in EPM. The psychopharmacological study of glutamatergic drugs revealed that memantine and lamotrigine, at all doses tested, decreased compulsive water consumption in HD rats compared to LD rats on SIP. NAC did not produce any significant effect on SIP. These results indicate that the symptom clusters of different forms of compulsivity and phobia might be found in the compulsive phenotype of HD rats selected by SIP. The effects of memantine and lamotrigine in HD rats point towards a dysregulation in the glutamatergic signaling as a possible underlying mechanism in the vulnerability to compulsive behavior on SIP. Further studies on SIP, could help to elucidate the therapeutic role of glutamatergic drugs as a pharmacological strategy on compulsive spectrum disorders.

## Introduction

Compulsivity has been defined as “the performance of repetitive, unwanted and functionally impairing overt or covert behavior without adaptive function according to either rigid rules or as a means of avoiding perceived negative consequences” (Fineberg et al., 2014). It is one of the principal symptoms in obsessive-compulsive disorder (OCD), that affects 2%–3% of the population and is considered as one of the ten leading neuropsychiatric disorders of disability (WHO, 2018). In the Diagnostic and Statistical Manual of Mental Disorders (5th edn), the *obsessive-compulsive and related disorders family* state that the course of OCD is often complicated by the co-occurrence of other disorders, including anxiety, specific phobia, depression, bipolar disorder, schizophrenia, and eating disorders as common comorbid pathologies (DSM-5; American Psychiatric Association, 2013). Indeed, compulsive behavior has been proposed as a trans-diagnostic symptom being comorbid especially with general anxiety disorders and depression (Gillan et al., 2017). For example, Torres et al. (2014, 2016) found that OCD patients, evaluated using the Dimensional Yale-Brown Obsessive–Compulsive Scale and Structured Clinical Interview for DSM-IV-TR Axis I Disorders, presented a lifetime prevalence of: 15.3% panic disorder (Torres et al., 2014), 56.4% major depression, 34.6% social phobia, 34.3% generalized anxiety disorder, and 31.4% specific phobia (Torres et al., 2016). Despite these studies, there are few experimental approaches in animals that have characterized the comorbidity with other altered pathological behaviors in preclinical models of compulsivity.

The clinical treatment of compulsivity in OCD patients has been focused on Serotonin reuptake inhibitors (SRIs), such as fluvoxamine, fluoxetine, sertraline, paroxetine and citalopram (reviewed in Fineberg and Gale, 2005). However, recent studies point out that up to 40% of patients do not respond successfully to SRIs treatment (Marinova et al., 2017). Recent studies suggest that glutamate-modulating drugs seem to have a beneficial effect in reducing compulsive symptoms in humans (Marinova et al., 2017) maybe because of its fundamental role in neuronal plasticity, learning, and memory (Javitt et al., 2011). Glutamate, the major excitatory neurotransmitter in the brain, is highly implicated in the cortico-striatal-thalamic circuit (Ting and Feng, 2011), the proposed neuroanatomical basis in compulsive deficit (reviewed in Menzies et al., 2008; Fineberg et al., 2010); which present a rich glutamatergic receptor density (Monaghan et al., 1985). A dysregulation of glutamatergic signaling in the cortico-striatal circuitry has been suggested in OCD, with reduced glutamatergic concentrations in the anterior cingulate cortex, as well as overactivity of glutamatergic signaling in the striatum and orbitofrontal cortex (Pittenger et al., 2011; Ting and Feng, 2011; Milad and Rauch, 2012).

Preclinical and clinical data have shown evidence that glutamatergic drugs could be a promising potential benefit in compulsive disorders. The N-Acetylcysteine (NAC), glutathione (GSH) precursor and a cell-permeable antioxidant, decrease the synaptic glutamate release (Moran et al., 2005). In clinical studies, NAC treatment has been shown to be effective in SRI-resistant OCD patients (Lafleur et al., 2006). Chronic treatment of NAC in OCD patients, 10–12 weeks, reduced the Yale-Brown Obsessive-Compulsive Scale (Y-BOCS; Afshar et al., 2012; Paydary et al., 2016). Moreover, it has also shown to improve symptomatology in other psychiatric syndromes, including depression, bipolar disorder, suicidality, and self-injurious behavior (Pittenger et al., 2005; Price et al., 2009; Niciu et al., 2014). In a preclinical study using an acute administration of 100 mg/kg of NAC reduced ethanol self-administration and ethanol-seeking behavior (Lebourgeois et al., 2018). Furthermore, memantine (MEM), an uncompetitive N-Methyl-D-aspartate (NMDA) receptor antagonist, that is currently employed in the treatment of Alzheimer disease (Reisberg et al., 2003) has also shown a beneficial effect in compulsivity. MEM reduce glutamate release through inhibition of voltage-dependent calcium channel and protein kinase C (Lu et al., 2010). In OCD patients, MEM reduced the Y-BOCS scores after chronic treatment with MEM (Ghaleiha et al., 2013; Haghighi et al., 2013). Preclinical studies showed that acute administration of 25 mg/kg MEM suppressed ethanol self-administration in non-dependent rats and decreased by half the one of post-dependent rats during acute withdrawal (Alaux-Cantin et al., 2015). Besides, the administration of MEM (10 mg/kg) and amantadine, another uncompetitive NMDA receptor antagonists (30 mg/kg), significantly inhibited compulsive marble burying in mice (Egashira et al., 2008). Moreover, the combination of MEM and fluoxetine reduced scratching behavior, considered as an effective model for studying compulsive behavior (Wald et al., 2009). Lamotrigine (LAM) is an established anticonvulsant drug, with antiepileptic activity due to the inhibition of the voltage-sensitive neuronal membrane sodium channels, inhibition of the excitatory amino acids release such as glutamate and aspartate, and blockade of the calcium-channel (Cheung et al., 1992; Xie et al., 1995; Cunningham and Jones, 2000; Prabhavalkar et al., 2015). A clinical study with chronic treatment with LAM evidenced a decrease in Y-BOCS scores in OCD patients, in addition to the Hamilton Rating Scale for Depression scores, the Clinical Global Impression-Improvement scores and the obsession and compulsion subscales (Bruno et al., 2012; Khalkhali et al., 2016). Besides, preclinical research showed that 15 and 30 mg/kg acute treatment of LAM significantly reduced immobility in the forced swimming test (FST; Li et al., 2010). However, there is insufficient preclinical research on the therapeutic role of these glutamate release modulators on reducing compulsive behaviors.

Schedule-induced polydipsia (SIP), a model of compulsive behavior (Moreno and Flores, 2012), is characterized by the development of an adjunctive behavior of repetitive drinking in food-deprived animals which are exposed to intermittent food-reinforcement schedules (Falk, 1961, 1966). An analogous phenomenon, called psychogenic polydipsia, which involves compulsive non-regulatory fluid consumption, is observed in 6%–20% of psychiatric patients (Evenson et al., 1987; de Leon et al., 1994, 2002; Dundas et al., 2007; Iftene et al., 2013). SIP is considered an animal model of compulsive drinking effective for studying the compulsive phenotype and modeling different psychopathologies related to compulsive spectrum disorders (Moreno and Flores, 2012; Hawken and Beninger, 2014; Belin-Rauscent et al., 2016). The individual differences observed on SIP acquisition support the selection of high compulsive drinking rats (HD) vs. low drinker rats (LD). In our laboratory, we have found consistent differences between these two populations in the inhibitory response deficit. Thus, HD rats selected by SIP have shown increased perseverative–compulsive responses under extinction conditions on the 5-Choice Serial Reaction Time task (5-CSRT; Moreno et al., 2012); impulsive decision making on the delay-discounting task (Cardona et al., 2011); less latent inhibition effect, considered as a behavioral model of schizophrenia, and augmented behavioral inflexibility in a spatial reversal learning task, characteristic in OCD patients (Navarro et al., 2017). Thus, HD and LD rats selected by SIP has shown consistent behavioral differences among different behavioral paradigms. Otherwise, SIP is considered a good model for researching the psychopharmacology of the compulsive phenotype (Platt et al., 2008; Moreno and Flores, 2012; Rodriguez et al., 2017). Indeed, studies on SIP revealed the efficacy of antipsychotic (haloperidol, clozapine, and pimozide) and antidepressant (fluoxetine) drugs in reducing SIP water intake (Snodgrass and Allen, 1989; Didriksen et al., 1993; Mittleman et al., 1994; Hogg and Dalvi, 2004; Dwyer et al., 2010). In HD rats selected by SIP, citalopram and the serotonin 5-HT_2A/C_ receptor agonist DOI reduced compulsive drinking (Navarro et al., 2017). Moreover, a recent study has revealed that HD rats showed cortical reduced serotonin 5-HT_2A_ receptor binding and increased serotonin and reduced glutamate efflux compared to LD rats (Mora et al., 2018). Therefore, the study of comorbid altered behaviors and the effect of glutamatergic drugs in compulsive HD rats selected by SIP could help for a better characterization of the compulsive endophenotype and explore new possible pharmacological targets for its treatment.

According to the previous clinical data, in the present study, first, we have explored the presence of other altered behaviors, including other forms of compulsivity and typical comorbid symptoms, such as depression, general anxiety and pathological fear disorder in the high compulsive drinker rats HD selected by SIP. The animal models selected to achieve this goal has been: the marble burying test (MBT) as a assay of compulsive-like behavior (Egashira et al., 2008; de Brouwer and Wolmarans, 2018); the FST developed in Porsolt et al. (1977) as an animal model of depression that assess learned helplessness; the elevated plus maze test (EPM) as a behavioral measure of anxiety for rodents (Pellow et al., 1985); and finally, the fear conditioning (FC) to test aversive learning considered as a behavioral paradigm that model specific phobias (Berardi et al., 2012). Furthermore, as a second goal, we assessed the efficacy of different glutamatergic drugs in reducing compulsive drinking on SIP. We explored the dose-response effects of acute administration of NAC, MEM, and LAM in reducing compulsive drinking on SIP. The results are discussed regarding the contributions of the characterization of comorbid altered behaviors in the compulsive phenotype rat population HD selected by SIP and the implication of the glutamatergic modulators as a new pharmacological strategy for compulsive neuropsychiatric disorders.

## Materials and Methods

### Subjects

A total of 16 male Long Evans rats (Janvier Labs, Le Genest-Saint-Isle, France) weighing between 250–350 g at the start of the experiments were used in the present study. The animals were housed four rats per cage (50 × 15 × 25 cm) at 22°C, with a 12:12-h light-dark cycle (lights off at 08:00 h) and food and water provided *ad libitum*. Before SIP training and after 10 days of habituation, rats were gradually reduced to 85% of their free-feeding body weight through controlled feeding, and their body weights were maintained at this level of deprivation throughout the experiments. Food was provided daily 30 min after each experimental session. All testing was performed between 9:00 and 15:00 h. All the procedures were conducted following the Spanish Royal Decree 53/2013 on the protection of experimental animals, the European Community Directive (2010/63/EU) for animal experiments and approved by the University of Almería Animal Research Committee.

### SIP Procedure

A complete description of the SIP procedure has been previously described (Moreno and Flores, 2012). First, over two successive days, we assessed the amount of water consumed by each rat in 60 min (baseline). Unlimited access to a bottle of water was provided (100 ml), and 60 food reward pellets were placed together (45 mg of dustless pellets; catalog number 259901-PE-45/50T TSE Systems, Germany). After one session of habituation to the SIP chambers (35 × 25 × 34 cm), the animals were exposed to a fixed time 60-s (FT-60s) schedule of food reward pellet presentation for 60-min sessions. During each SIP session, a bottle of water (100 ml) was positioned opposite the food-magazine in the SIP chamber, the amount of water intake was recorded at the end of the test session. The licking behavior to the bottle of water was detected when the animal touches the metal drinking tube (spout) of the bottle. The spout is connected to the metal grid of the SIP chamber, where the animal stands, by an electronic circuit with a low current, less than ten microAmp, inappreciable to the animal. When the rat touches the water spout of the bottle, this closes the circuit, producing a 50 ms pulse, which registers a lick. The scheduling and recording of the experimental events are controlled using a computer and the commercial software Med PC (Cibertec SA, Madrid, Spain). For each rat, we recorded the following measures: the total amount of water (milliliters) removed from the bottle, the total number of licks to the bottle, and the total entries to the food magazine. After 20 daily sessions, the animals were separated into two specific populations, HD and LD, according to whether their rates of drinking (average for each animal over the last five sessions) were above or below the group median, respectively (the number of animals in each group of LD and HD rats was *n* = 8).

### Experimental Design

**Figure 1:**
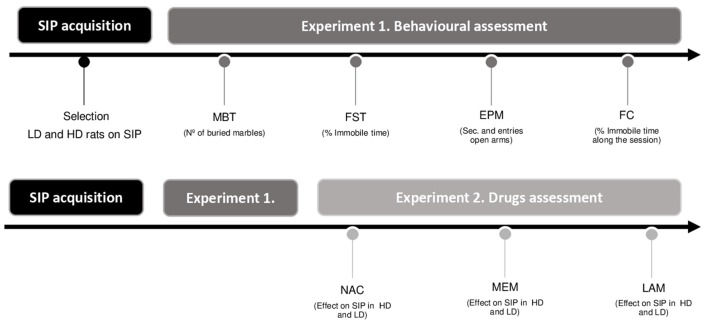
Experimental procedure illustrated in a timetable (SIP, schedule-induced polydipsia; LD, low drinkers; HD, high drinkers; MBT, marble burying test; FST, forced swimming test; EPM, elevated plus maze test; FC, fear conditioning test; NAC, N-Acetylcysteine; MEM, memantine; and LAM, lamotrigine).

The order of the behavioral assessment and drug testing are summarized in Figure 1.

#### Experiment 1

##### Behavioral Assessment

We examined the presence of other altered behaviors considered as comorbid symptoms for compulsivity in high compulsive animals selected by SIP. We assessed compulsive-like behavior on MBT (Taylor et al., 2017), depressive-like behavior on FST (Yan et al., 2010), anxiety-like behavior on EPM (Pellow et al., 1985) and specific phobia behavior on FC (Berardi et al., 2012) in LD and HD rats selected by SIP (*n* per group = 8). The screening in each test commenced at least 1 week after the previous one.

#### Experiment 2

##### Glutamatergic Drugs

The behavioral effects of acute systemic administration of different glutamatergic drugs were tested in both groups of LD and HD rats in SIP (*n* per group = 8). We explored the effects of acute intraperitoneal injections (i.p.) of NAC (25, 50, 100 and 200 mg/kg), MEM (3.1 and 6.2 mg/kg) and LAM (15 and 30 mg/kg) in LD and HD rats in SIP. The drug doses, the injection time of 60 min before behavioral testing, were selected based on previous experiments (Li et al., 2010; Réus et al., 2010; Lebourgeois et al., 2018). All animals received drugs according to a fully randomized Latin-square design, separated by a minimum of 72 h between drug test sessions. There was a wash-out period of 1 week between each drug tested (animals continued performing SIP sessions during this week). The experimental sessions were led on Tuesdays and Fridays, and baseline testing was accomplished on Mondays and Thursdays. On Wednesdays, animals performed SIP procedure, but the results were not analyzed.

##### Behavioral Assessment

MBT began placing the rat into a corner of the cage containing nine marbles, being careful to place the rat on bedding as far from marbles as possible. Animals were allowed to remain in the cage undisturbed for 30 min. Rats were returned to its home cage after test completion, taking extreme care not to move or dislodge the marbles in the process of removing the subject from the cage. The number of marbles partially and completely buried was counted by two observers blinded to the experimental groups. We found a great concordance between observers. A marble was scored as partially buried if two-thirds of its surface area is covered by bedding and completely buried if all the surface area is covered by bedding (Angoa-Pérez et al., 2013).

FST was performed in a plastic cylinder containing 20 cm in diameter and 40 cm in height water temperature was 23–25°C, and the depth of water was set to prevent animals from touching the bottom. Rats swam in the cylinder for 2 min. The time each animal spent immobile during the last min of the test was counted by two observers blinded to the experimental groups. We found a great concordance between observers. Immobility was defined as floating or absolute lack of motion (i.e., the absence of all movements except those required to maintain balance; Dong et al., 2018).

For EPM rats were placed at the junction of the four arms of the maze, facing an open arm, and entries/duration in each arm was recorded by a video-tracking system and observer simultaneously for 10 min. We found a good concordance in data collected with both methods. An increase in open arm activity (duration and/or entries) reflects anti-anxiety behavior (Walf and Frye, 2007).

FC started placing the rat into a novel set of cages with a shock grid floor capable of delivering foot-shock where, after 3 min exploration period, they received three pairings of a 10 s light (82 lx) with a shock (0.5 mA during 1 s). The light-shock trials were delivered after a 3-min acclimation time, the inter-lights intervals were 1 min, and the rats remained in the chambers for an additional minute after the last shock. Next day rats were allowed a 3 min exploration period after which they were presented with 22 lights (10 s, 82 lx, 1 min inter-lights interval) in the absence of a foot shock (Simone and McCormick, 2017). The freezing time was counted by the Video Freeze Software (Med PC) which detected changes at the pixel level from one video frame to the next. Hence, data can reflect the total time animals spent in motionless during the session, the percentage of time motionless and the number of freezing episodes.

### Drugs

We explored the effects of acute intraperitoneal injections (i.p.) of NAC (25, 50, 100 and 200 mg/kg; Lebourgeois et al., 2018), MEM (3.1 and 6.2 mg/kg; Li et al., 2010) and LAM (15 and 30 mg/kg; Réus et al., 2010) in LD and HD rats in SIP. NAC [(2R)-2-(Acetylamino)-3-mercapto propanamide] and MEM [3, 5-Dimethyl-tricyclo (3.3.1.13, 7) decan-1-amine hydrochloride] were dissolved in 0.9% saline. LAM [6-(2, 3-Dichlorophenyl)-1, 2, 4-triazine-3, 5-diamine] was suspended in 1% Tween-80 in 0.9% saline. All drugs were purchased from Sigma-Aldrich (Madrid, Spain). The injection volumes were 1 ml/kg for all drugs. For all drug solutions, the final pH was adjusted to approximately 6.4 using 0.1 M NaOH, and they were aliquoted after preparation and frozen at −80°C before use.

### Data Analyses

Behavioral data on SIP acquisition were analyzed using two-way repeated-measure analysis of variance (ANOVA), with “group” (LD and HD) as the between-subject factor and “sessions” (20 sessions) as the within-subject factor. The differences on the MBT, FST, EPM, and FC of the behavioral assessment in LD and HD were studied using Student’s *t*-test (*T*-test). When appropriate, the effect size of the group differences was calculated using Cohen’s d (*d*; mean difference divided by pooled standard deviation). The differences on FC blocks and the effects of the different drugs in LD and HD on SIP were analyzed using two-way repeated-measure ANOVA, with group (LD and HD) as the between-subject factor and “percentage of freezing” (percentage of time spent on freezing during the different blocks of the retrieval day) or “drug” (different doses of drug and vehicle) as the repeated within-subject factor. When appropriate, the effect size of the group differences was calculated using eta-squared (*η*^2^). *Post hoc* comparisons were performed using the Newman-Keuls test. Statistical significance was set at *p* < 0.05. All analyses were computed using Statistica software (version 6.0).

## Results

### LD and HD Selected by SIP

**Figure 2:**
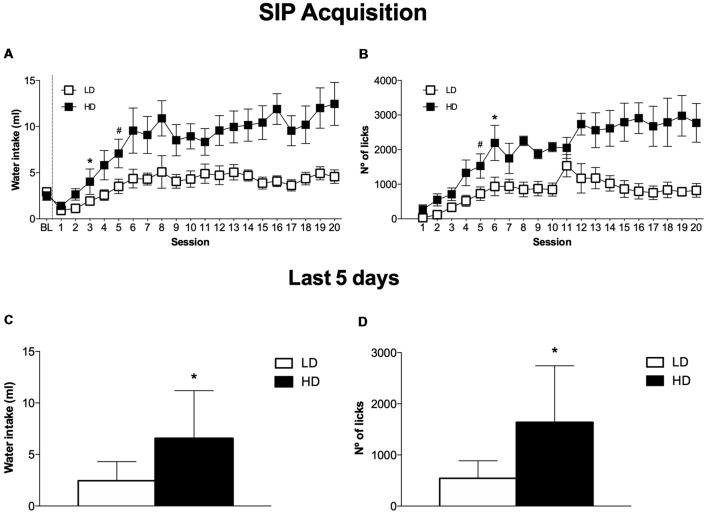
The mean (± SEM) water intake **(A)** and number of licks **(B)** in FT-60s across 20 sessions of SIP. The mean (± SEM) water intake **(C)** and number of licks **(D)** in FT-60s across the last five sessions of SIP acquisition. Statistical analyses indicate significant differences between low drinkers (LD, *n* = 8) and high drinkers (HD, *n* = 8; *p < 0.05). Significant differences between sessions were found from session 1 (^#^*p* < 0.05).

The mean water intake and licks in LD and HD during the acquisition and maintenance of SIP is shown in Figures 2A,B. In the experimental phase, the mean water intake over the last 5 days of SIP was 4.3 ± 0.6 and 11.2 ± 1.9 ml for LD and HD, respectively (Figure 2C). The number of licks also showed SIP acquisition. The mean total licks averaged across the last 5 days of SIP were 885.1 ± 202.9 and 2742.9 ± 536.9 for LD and HD, respectively (Figure 2D).

ANOVA revealed significant differences in water intake according to the interaction between SIP acquisition sessions and LD vs. HD (SIP session effect: *F*_(19,266)_ = 11.759, *p* < 0.001; group effect: *F*_(1,14)_ = 10.332, *p* < 0.01; interaction SIP session × group effect: *F*_(19,266)_ = 2.58, *p* < 0.001). This difference was also confirmed by the significant interaction observed in the total number of licks (SIP session effect: *F*_(19,266)_ = 11.890, *p* < 0.001; group effect: *F*_(1,14)_ = 13.647, *p* < 0.01; interaction SIP session × group effect: *F*_(19,266)_ = 3.38, *p* < 0.001). *Post hoc* analysis indicated significant differences between the LD and HD animals in the water intake at session 6 (*p* < 0.01) onwards. Furthermore, animals in the HD group significantly increased their consumption of water from session 4 (*p* < 0.05) compared to session 1. Differences between the LD and HD groups in the number of total licks at session 6 (*p* < 0.05) were also observed, and HD rats increased their number of licks from session 5 (*p* < 0.001) compared to session 1. We also found significant differences in the number of magazine entries according to the interaction between SIP acquisition sessions and LD vs. HD (session × group effect: *F*_(19,266)_ = 2.124; *p* < 0.01; session effect: *F*_(19,266)_ = 4.515, *p* < 0.001; group effect: *F*_(1,14)_ = 5.577, *p* < 0.05). Differences between the LD and HD groups in the number magazine entries at session 11 (*p* < 0.001 were also observed, and HD rats increased their number of magazine entries from session 6 (*p* < 0.05) compared to session 1.

### Experiment 1

#### Behavioral Assessment

##### Marble Burying Test

**Figure 3:**
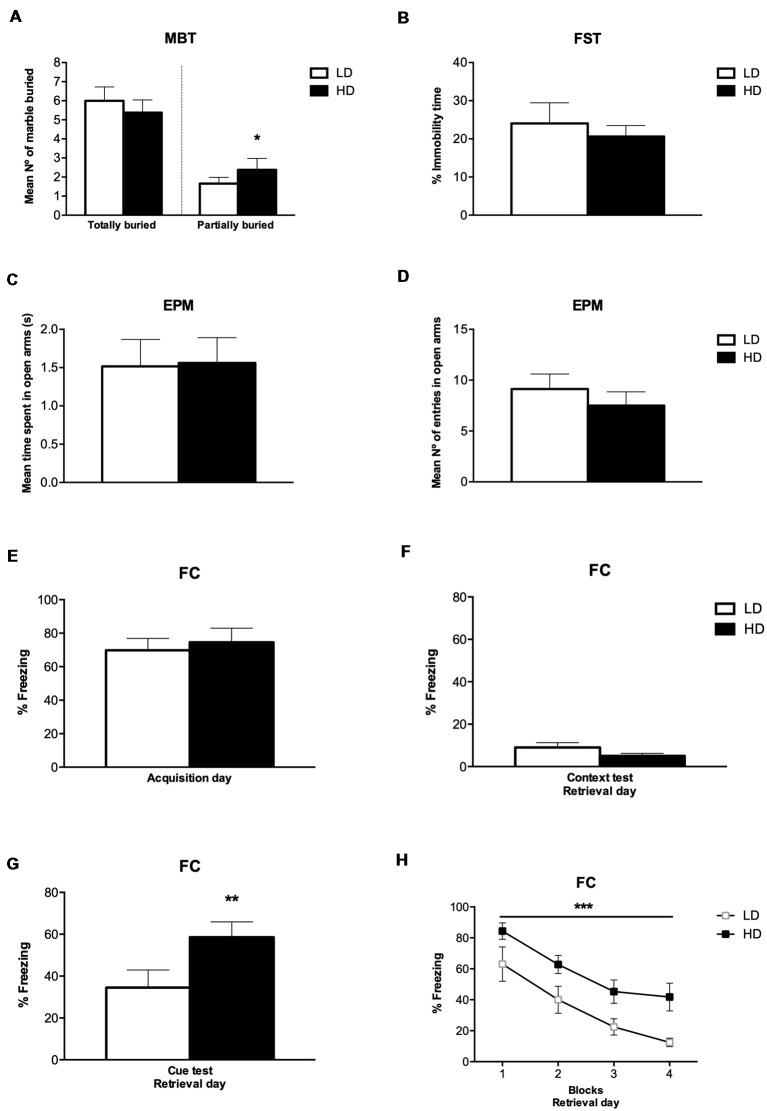
Behavioral assessment after SIP procedure. **(A)** MBT scores of low drinkers (LD, *n* = 8) and high drinkers (HD, *n* = 8) rats, **(B)** percentage of immobile time LD and HD rats spent on forced swimming test (FST), **(C)** mean number of entries by LD and HD rats on the open arms in elevated plus maze test (EPM), **(D)** seconds spent by LD and HD rats on the open arms in EPM, **(E)** percentage of freezing LD and HD rats exhibited during fear acquisition day, **(F)** percentage of freezing LD and HD rats exhibited during contextual fear test on retrieval day, **(G)** percentage of freezing LD and HD rats exhibited during cued fear test on retrieval day, and **(H)** percentage of freezing LD and HD rats exhibited during the four blocks of time (6 min per block) at cued fear test on retrieval day of fear conditioning procedure (FC). Data are expressed as the means ± SEM. *p < 0.05; **p < 0.01; ***p < 0.001 indicate significant differences between groups.

The number of marbles partially (2/3) and completely buried by LD and HD rats on MBT are shown in Figure 3A. *T*-test and the effect sizes by Cohen’s d showed that HD rats had a significantly increased number of marbles partially (2/3) buried compared to LD rats (*df* = 14; *T*-test = −2.22; *p* < 0.05; *d* = 1.186). There was no significant effect on the number of marbles completely buried between LD and HD rats (*df* = 14; *T*-test = 1.14; *p* = 0.27).

##### Forced Swimming Test

The percentage of immobile time of LD and HD rats on FST are shown in Figure 3B. *T*-test showed no significant difference in the percentage of immobile time between LD and HD rats (*df* = 14; *T*-test = 0.35; *p* = 0.72).

##### Elevated Plus Maze Test

The time LD and HD rats spent on the open arm before changing to the other, and the number of entries in the open arm on EPM are shown in Figures 3C,D. *T*-test showed that there was no significant difference in the mean time and the number of entries in the open arms between LD and HD rats (*df* = 14; *T*-test = −0.09; *p* = 0.92; *df* = 14; *T*-test = 0.86; *p* = 0.40). The mean time LD and HD rats spent on the closed arm before changing to the other was 1.53 ± 0.35 and 1.83 ± 0.39, respectively. The mean number of entries in the closed arm on EPM was 9.38 ± 0.67 for LD rats and 8.88 ± 1.24 for HD rats. *T*-test showed that there was no significant difference in the mean time and the number of entries in the closed arms between LD and HD rats (*df* = 14; *T*-test = −0.60; *p* = 0.56; *df* = 14; *T*-test = 0.38; *p* = 0.71). The mean time LD and HD rats spent on one arm before changing to another one was 1.53 ± 0.06 and 1.70 ± 0.08, respectively. The mean number of entries in open and closed arms was 18.50 ± 1.14 for LD rats and 16.38 ± 1.23 for HD rats. *T*-test showed that there was no significant difference in the mean time and the number of entries in open and closed arms between LD and HD rats (*df* = 14; *T*-test = −1.85; *p* = 0.08; *df* = 14; *T*-test = 1.35; *p* = 0.20).

##### Fear Conditioning

The percentage of freezing time of LD and HD rats on FC during the acquisition day, the percentage of freezing time during the contextual fear test and the cued fear test at the retrieval day, as well as the percentage of freezing during the different blocks of trials on the retrieval day, is shown in Figures 3E–H. No significant differences were found in the percentage of freezing time spent by LD and HD rats during the acquisition day (*df* = 14; *T*-test = −0.45; *p* = 0.65), nor in the contextual fear test on the retrieval day (*df* = 14; *T*-test = −1.51; *p* = 0.15). However, *T*-test and effect sizes by Cohen’s d revealed a significant increase in the percentage of freezing time spent by HD compared to LD rats during the cue presentation on retrieval day (*df* = 14; *T*-test = −3.12; *p* < 0.01; *d* = 1.67). The analyses of the 4 blocks of trials on the retrieval day by ANOVA and *η*^2^ revealed that both, LD and HD rats, significantly reduced the percentage of freezing time in the different blocks of the retrieval day (Trial effect: *F*_(3,42)_ = 36.64; *p* < 0.001; *η*^2^ = 0.931); whether the significant increased percentage of freezing time spent by HD compared to LD rats was maintained through the four blocks of trials on the retrieval day (group effect: *F*_(1,14)_ = 9.73; *p* < 0.01; *η*^2^ = 0.933). No significant differences were observed by group × trial interaction (*F*_(3,42)_ = 0.27; *p* = 0.84).

## Experiment 2

### Glutamatergic Drugs

#### N-Acetylcysteine

**Figure 4:**
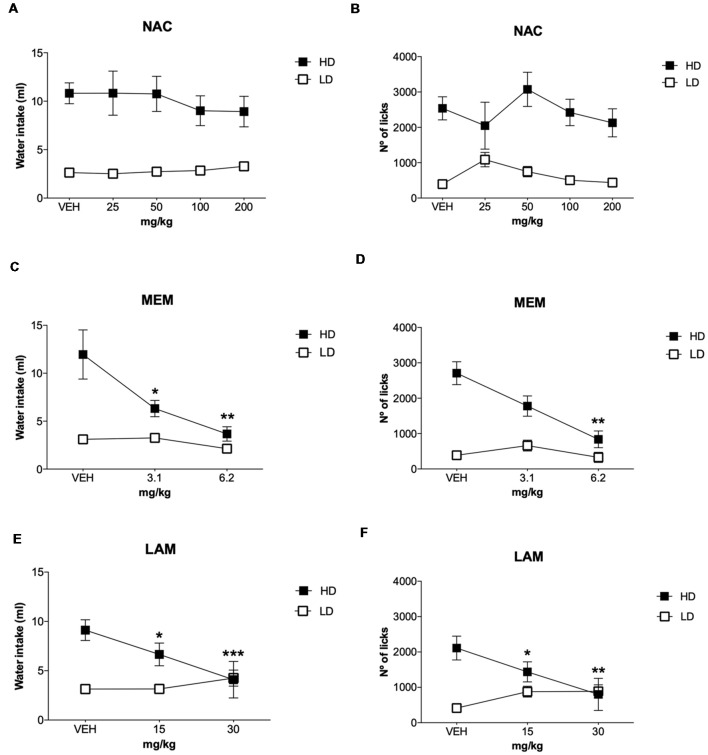
Glutamatergic drugs on SIP. Effects of **(A,B)** NAC, **(C,D)** memantine (MEM), and **(E,F)** lamotrigine (LAM) administration on water intake and number of licks in low drinkers (LD, *n* = 8) and high drinkers (HD, *n* = 8) rats on SIP. Data are expressed as the means ± SEM. *p < 0.05; **p < 0.01; ***p < 0.001 indicate significant differences vs. vehicle administration in the same group of rats.

The effects of NAC on water intake and licks in SIP are shown in Figures 4A,B, and the number of magazine entries after NAC administration are shown in Table 1. ANOVA showed that NAC did not induce significant differences in water intake (group × drug interaction, *F*_(4,56)_ = 0.63, *p* = 0.64; group effect, *F*_(1,14)_ = 109.15, *p* < 0.001; drug effect, *F*_(4,56)_ = 0.38, *p* = 0.82), total licks (group × drug interaction, *F*_(4,56)_ = 0.57, *p* = 0.68; group effect, *F*_(1,14)_ = 111.89, *p* < 0.001 drug effect, *F*_(4,56)_ = 0.90, *p* = 0.47), and magazine entries (group × drug interaction, *F*_(4,56)_ = 0.28, *p* = 0.89; group effect, *F*_(1,14)_ = 8.41, *p* < 0.05; drug effect, *F*_(4,56)_ = 0.51, *p* = 0.73).

**Table 1:** Effects of N-Acetylcysteine (NAC), memantine (MEM) and lamotrigine (LAM) on total magazine entries in low drinkers (LD, *n* = 8) and high drinkers (HD, *n* = 8) rats on schedule-induced polydipsia (SIP).

	Total magazine entries	
	LD	HD
**N-Acetylcysteine**		
Vehicle	996.59 ± 126.34	2,052.68 ± 314.41
25 mg/kg	1,087.19 ± 205.43	2,047.67 ± 664.21
50 mg/kg	1,011.57 ± 162.58	2,041.77 ± 488.66
100 mg/kg	987.75 ± 199.97	2,095.57 ± 676.96
200 mg/kg	1,006.29 ± 183.00	1,428.29 ± 247.77
**Memantine**		
Vehicle	961.13 ± 144.41	1,584.11 ± 206.60
3.1 mg/kg	992.71 ± 197.17	1,173.43 ± 116.98
6.2 mg/kg	930.00 ± 222.24	811.67 ± 182.63
**Lamotrigine**		
Vehicle	1,058.71 ± 134.80	1,192.29 ± 111.46
15 mg/kg	1,093.57 ± 152.87	1,286.14 ± 145.31
30 mg/kg	1,135.69 ± 233.17	554.27 ± 162.55*

Data are expressed as the means ± SEM. *p < 0.05 indicate significant differences vs. vehicle administration in the same group of rats.

#### Memantine

The effects of MEM on water intake and total licks in SIP are shown in Figures 4C,D. Effects of MEM on magazine entries are depicted in Table 1. MEM significantly reduced compulsive water intake in HD rats compared to LD rats (group × drug interaction, *F*_(2,28)_ = 4.51, *p* < 0.05; group effect, *F*_(1,14)_ = 24.05, *p* < 0.001; drug effect, *F*_(2,28)_ = 8.42, *p* < 0.01; *η*^2^ = 0.930). *Post hoc* analyses revealed that MEM reduced dose-dependent water intake in HD rats at both doses: 3.1 (*p* < 0.05) and 6.2 mg/kg (*p* < 0.001) compared with vehicle in the same group. MEM did not affect water intake in LD rats. The comparison between LD and HD revealed a dose dependent reduction of the significant differences in water intake disappearing at the highest dose (vehicle, *p* = 0.0001; 3.1 mg/kg, *p* = 0.041; 6.2, *p* = 0.572). Moreover, MEM also significantly reduced the total licks in HD rats compared with the LD group (group × drug interaction, *F*_(2,28)_ = 6.04, *p* < 0.01; group effect, *F*_(1,14)_ = 16.96, *p* < 0.05; drug effect, *F*_(2,28)_ = 5.50, *p* < 0.01; *η*^2^ = 0.730). *Post hoc* comparison confirmed a decrease in the total licks in the HD group at the highest dose used 6.2 mg/kg (*p* < 0.001) compared with vehicle in the same group. Differences between LD and HD remained significant at all doses tested. MEM administration did not affect the number of magazine entries in both groups of rats (group × drug interaction: *F*_(2,28)_ = 2.663; *p* = 0.087; drug effect: *F*_(2,28)_ = 2.507; *p* = 0.099; group effect: *F*_(1,14)_ = 1.569; *p* = 0.23).

#### Lamotrigine

The effects of LAM on water intake and total licks in SIP are shown in Figures 4E,F. The effects of LAM on magazine entries in SIP are shown in Table 1. LAM significantly reduced compulsive water intake in HD rats compared to LD rats (group × drug interaction: *F*_(2,28)_ = 11.396, *p* < 0.0002; group effect: *F*_(1,14)_ = 5.187, *p* < 0.05; drug effect: *F*_(2,28)_ = 3.532, *p* < 0.05; *η*^2^ = 0.882). *Post hoc* analyses revealed that LAM reduced dose-dependent water intake in HD rats at both doses: 15 (*p* < 0.05) and 30 mg/kg (*p* < 0.01) compared with vehicle in the same group. LAM reversed the significant differences on water intake between LD and HD rats on SIP (vehicle, *p* = 0.008; 15 mg/kg *p* = 0.16; 30 mg/kg, *p* = 0.914). LAM did not affect water intake in LD rats. Moreover, LAM also significantly reduced the total licks in HD rats compared with the LD group (group × drug interaction, *F*_(2,28)_ = 11.40, *p* < 0.001; group effect, *F*_(1,14)_ = 5.18, *p* < 0.05; drug effect, *F*_(2,28)_ = 3.53, *p* < 0.05; *η*^2^ = 0.870). *Post hoc* comparison showed a dose dependent decrease in the total licks in the HD group at both doses used 15 mg/kg (*p* < 0.05) and 30 mg/kg (*p* < 0.001) compared with vehicle in the same group. The comparison between LD and HD revealed a dose dependent reduction of the significant differences in the number of licks disappearing at the highest dose (vehicle, *p* = 0.0001; 15 mg/kg *p* = 0.005; 30 mg/kg, *p* = 0.86). LAM administration reduced magazine entries in both groups of rats (group × drug interaction: *F*_(2,28)_ = 3.61, *p* < 0.05; group effect: *F*_(1,14)_ = 0.19, *p* = 0.67; drug effect: *F*_(2,28)_ = 4.65, *p* < 0.05; 0.931). *Post hoc* analyses revealed a decrease in magazine entries in HD rats only at the highest dose tested 30 mg/kg (*p* < 0.05) compared with vehicle and with the LD group.

## Discussion

The present study investigated the presence of possible comorbid symptoms (compulsive, depressive, anxious and fear behavior) in animals selected by high compulsive drinking behavior on SIP, HD rats. Moreover, we investigated the therapeutic potential of glutamatergic drugs for reducing compulsive drinking behavior in HD rats on SIP. The findings showed that HD rats, characterized by excessive and persistent compulsive drinking on SIP, also exhibited a compulsive behavior on MBT by a higher number of marbles partially buried (2/3) compared to LD rats. Besides, compulsive HD rats selected by SIP had an increased fear behavior profile on FC, showed by a higher percentage of freezing time in the first block of the retrieval day as well as across the following blocks, compared to LD rats. These differences between HD and LD rats might not be attributed to individual differences in reactivity to novelty. HD rats selected by SIP did not differ in spontaneous locomotor reactivity to novelty compared with LD rats (Moreno et al., 2012). Moreover, in the present study, no significant differences were found in the number of magazine entries, considered as a control measure of motor activity or motivational behavior (Navarro et al., 2015), between HD and LD on SIP.

The acute administration of glutamatergic drugs revealed that MEM and LAM reduced, in a dose-dependent manner, compulsive intake in HD rats on SIP, and did not affect LD behavior. Hence, the observed effect cannot be considered as a compensatory behavior by the use of these treatments. Moreover, we discard other possible side effects, as in previous studies the selected doses of NAC, MEM, and LAM did not affect locomotor activity in rats (Li et al., 2010; Réus et al., 2010; Lebourgeois et al., 2018). However, NAC administration did not selectively affect compulsive intake in SIP, as LD and HD kept significant differences at all doses administrated.

### Assessment of Comorbid Behaviors on Compulsive HD Rats

HD rats selected by SIP showed comorbidity with compulsive behavior on MBT, by a significantly increased number of marbles partially buried compared to LD rats. Previous studies have found that HD rats selected by SIP showed other behavioral compulsivity forms such as compulsive lever pressing, during the pre-training phase to assess latent inhibition (Navarro et al., 2017), proposed as an OCD model (Joel and Avisar, 2001); and behavioral inflexibility in a spatial reversal task (Navarro et al., 2017). In contrast, other studies on rats with high levels of grooming, considered as a compulsive-like behavior, have shown a reduced number of marbles buried in MBT, showing a negative correlation between these factors (Reimer et al., 2015). The reason for these contradictory results could be due to the fact that compulsivity is not a unitary phenomenon and can be expressed by different forms (Fineberg et al., 2018).

The assessment of depressive behavior revealed that LD and HD rats selected by SIP did not exhibit any differences in depressive-like behavior measured on FST. The compulsive HD rats might not have depression signs as a comorbid behavior. Nevertheless, other preclinical studies have shown associations between depressive and compulsive behavior in the same individuals. For example, the administration of 8-OH-DPAT, a 5-HT_1A_ agonist, proposed as an OCD model (Yadin et al., 1991), increased the immobility time on FST (Sela et al., 2010). Moreover, the administration of the purinergic receptor P2R antagonist [pyridoxalphosphate-6-azophenyl-2’, 4’-disulfonic acid tetrasodium salt (PPADS)] in Swiss mice, reduced depressive-like behavior in the FST, as well as compulsive-like behavior in MBT (Pereira et al., 2013). The effect of antidepressants on addictions, considered as compulsive disorders, has created some controversy. On the one hand, some preclinical studies have demonstrated reductions in alcohol addiction subsequently to the administration of different 5-HT receptors agonists (Naranjo et al., 1986; Higley et al., 1998; Martijena et al., 2005). On the other hand, the possibility that antidepressant treatment might increase susceptibility to alcoholism has been overlooked (Alén et al., 2013, 2014). Moreover, several clinical studies have shown that pathological gambling, associated with elevated compulsivity, frequently co-occurs with major depression (Cunningham-Williams and Cottler, 2001; Baer et al., 2015; Redden et al., 2015; Agarwal et al., 2016; Grant et al., 2016; Rickelt et al., 2016). More research is needed to clarify the relation between depressive and compulsive behavior.

Anxiety behavior measured by EPM did not show any significant differences between HD and LD rats selected by SIP. Nevertheless, we have replicated the results published in 2008 by López-Grancha, in which there were no differences in the EPM between LD and HD rats selected by SIP (López-Grancha et al., 2008). Moreover, animals with distinct levels of self-grooming emission, considered as a compulsive-like behavior, did not differ in the exploration of the EPM (Reimer et al., 2015). In contrast, a previous study has shown that an increased compulsive behavior in the MBT has also been accompanied by increased anxiety response in the EPM and open-field test in the same animals (Mitra et al., 2016). These contradictory results posit the relevance of the study on individual differences, using populations more prone to a behavioral deficit. Self-grooming and MBT might be evaluating different kinds of compulsivity, as well as anxiety is also a neuropsychological domain that could be expressed by different symptoms (reviewed in Ströhle et al., 2018). For instance, compulsive drinkers HD rats selected by SIP did not differ in anxiety-like behavior assessed using EPM to LD rats, while they differed in anxiety-like behavior measured by freezing time on the retrieval day in FC.

The assessment of fear behavior by FC revealed that HD rats selected by SIP showed a significantly augmented percentage of freezing time compared to LD rats during cued-fear memory on the retrieval day. Thus, HD and LD rats had no differences in the percentage of freezing time on the acquisition day, nor in the exploration period when exposed to the fear context on the retrieval day. Previous findings in our laboratory, have shown that under extinction conditions, HD rats had a greater increase in perseverative responses, considered as compulsive behavior, compared to LD rats on 5-CSRT (Moreno et al., 2012). Moreover, HD rats have shown increased c-Fos activity in the basolateral amygdala compared with LD rats (Merchán et al., 2019). The basolateral amygdala, as an essential structure in the neural system for FC (Phillips and LeDoux, 1992; Vazdarjanova and McGaugh, 1998), is highly implicated in cued-related fear memories and not essential for contextual FC (reviewed in Curzon et al., 2009). HD animals selected by SIP might be a convenient phenotype to study the neuronal basis of individual differences in habit formation under extinction conditions. Thus, in HD rats, a possible alteration in the basolateral amygdala might underlie the observed increased cued-fear memory on FC that possibly also affect the vulnerability to develop compulsive behaviors. In this sense, clinical studies demonstrated that OCD patients continued to exhibit a differential skin conductance response to the conditioned stimuli in the extinction phase of fear conditioned computer task, while control participants extinguished fear (Geller et al., 2017). Translational neuroscience studying fear could help us to better understand brain circuitry underlying fear behavior, although the translation of animal model results into the clinic is limited and more research is needed (Flores et al., 2018).

### Effects of Glutamatergic Drugs on Compulsive Rats on SIP

The administration of NAC (25, 50, 100 and 200 mg/kg) revealed no significant differences in the water intake nor LD, nor in HD rats on SIP. Conversely, previous research has demonstrated that NAC (90 mg/kg), chronically and systemically administered, resulted in significant reductions of compulsive binge eating in a rodent model (Hurley et al., 2016). NAC systemically administrated has been demonstrated to abolish the recovery of compulsive cocaine-seeking behavior in a rodent model through augmenting the glutamate/cystine antiporter activity and reestablishing the concentration of extracellular glutamate in the nucleus accumbens (Baker et al., 2003a,b). Moreover, the acute administration of NAC at 100 mg/kg reduced motivation, seeking and relapse to self-administration of ethanol in rats (Lebourgeois et al., 2018). However, acute injections of NAC (0, 30, 60, or 120 mg/kg) did not have any result on self-administration of methamphetamine in rats (Charntikov et al., 2018). Some clinical studies have suggested the possible therapeutic role of NAC in OCD patients, showing a reduction in the scores of the Y-BOCS after treatment with NAC during 10 and 12 weeks respectively (Afshar et al., 2012; Paydary et al., 2016).

The acute systemic administration of MEM, 3.1 and 6.2 mg/kg, decreased compulsive drinking in HD rats on SIP, compared to LD rats that remain unaffected. Hence, these results could not be considered as a general effect on rats exposed to SIP, pointing towards the neuropsychopharmacological effects of MEM might be involved in the vulnerability to compulsive non-regulatory drinking on SIP. In contrast, previous studies, have found that acute administration of MEM at 5 and 25 mg/kg in mice, did not affect water intake on SIP, but revealed a reduction in regulatory drinking (Escher et al., 2006). Although in this study, mice were not selected according to the rate of compulsive drinking. However, in the same study MEM have been found as a useful treatment for reducing compulsive alcohol intake, the administration of MEM 10 and 25 mg/kg significantly reduced alcohol drinking in mice on SIP (Escher et al., 2006). Moreover, findings revealed that acute administration of 10 mg/kg MEM significantly inhibited compulsive behavior in MBT without affecting locomotor activity in mice (Egashira et al., 2008). Furthermore, acute administration of 25 mg/kg MEM blocked ethanol self-administration in non-dependent rats, as well as it decreased by half the one of post-dependent rats during acute withdrawal (Alaux-Cantin et al., 2015). Otherwise, compulsive lever pressing, proposed as an OCD model (Joel and Avisar, 2001), was not affected by an NMDA antagonist (MK 801), while an NMDA partial agonist (D-cycloserine) decreased this behavior (Albelda et al., 2010). In this sense, the present results also contrast with the no effect found after ketamine administration in HD and LD rats on SIP (Martín-González et al., 2018). Though both ketamine and MEM typify the same kind of drugs, they diverge in voltage dependence and blocking kinetics (Danysz and Parsons, 1998). In human studies, MEM showed a therapeutic role in obsessive-compulsive patients, by reducing the Y-BOCS scores after chronic treatment with MEM during 8 weeks (Ghaleiha et al., 2013) and 12 weeks (Stewart et al., 2010; Haghighi et al., 2013). Other study investigating MEM augmentation of risperidone treatment in children with autism spectrum disorders revealed that the group receiving MEM showed significant improvements in the subscales: irritability, stereotypic behavior, and hyperactivity of the Aberrant Behavior Checklist-Community (Ghaleiha et al., 2013).

Our data showed that the administration of LAM, 15 and 30 mg/kg, significantly decreased compulsive water drinking in HD rats, compared to LD rats, on SIP. There are few preclinical studies on the behavioral effects of LAM, most of them related to as an anti-depressant like effect. The acute administration of LAM at 16 and 32 mg/kg of LAM induced a reduction in immobility time in the FST (Prica et al., 2008). Similarly, LAM at 15 and 30 mg/kg significantly reduced immobility in the FST (Li et al., 2010). In human studies, have evidenced that 16 weeks of treatment with LAM in obsessive-compulsive patients significantly reduced the Y-BOCS scores, as well as the Hamilton Rating Scale for Depression scores and the Clinical Global Impression-Severity scores (Bruno et al., 2012). More recently, two other studies using adjunctive treatment of LAM in addition to SRIs treatment led in treatment-resistant OCD patients during 8 and 12 weeks respectively, revealed a greater reduction in total YBOCS scores in LAM group (Hussain et al., 2015; Khalkhali et al., 2016).

Collectively, the beneficial effects of MEM and LAM administration in reducing compulsive drinking in HD rats on SIP suggest a therapeutic role for glutamate inhibition, antagonizing NMDA receptor or blocking calcium and sodium channels in pre-synaptic terminals. In contrast, the lack of effect of NAC in compulsive intake in HD rats on SIP posits the idea of the possible relevance of the differential effect by the specific stimulation of the presynaptic terminal. These results support the possible dysregulation in glutamatergic signal previously observed, in which HD rats selected by SIP showed a decreased basal level of glutamate in the medial prefrontal cortex (mPFC), restored by serotonin 5-HT_2A/C_ agonist DOI (Mora et al., 2018). Moreover, the effects of glutamatergic drugs MEM and LAM suggest a possible modulatory role in the neuroanatomic and neurochemical alterations observed in dopamine D_2_ receptors and 5-HT_2A_ receptors in HD rats selected by SIP (Pellón et al., 2011; Moreno et al., 2012; Mora et al., 2018).

Preclinical studies on compulsivity, using the dopamine D_2_ and D_3_ receptor agonist quinpirole (QNP) in rats (Szechtman et al., 1998), have also evidenced a dysregulation by an increased glutamate release in the subtantia nigra and a lower extracellular concentration in the nucleus accumbens (Abarca et al., 1995; Krügel et al., 2004; Escobar et al., 2015). Therefore, the proposed underlying mechanism in compulsivity of the QNP-OCD model was associated with decreased dopaminergic and glutamatergic neurotransmission in the mPFC to the nucleus accumbens, pointing toward a loss of executive control (Escobar et al., 2015). Furthermore, NMDA dependent glutamate neurotransmission in the cortico-striatal circuitry seems to play a central role by the functional interaction with serotonin and dopamine receptors in executive response control and compulsivity measured by the 5-CSRT (reviewed in Carli and Invernizzi, 2014). In example, the local infusions of NMDA receptor antagonist 3-((R)-2-carboxypiperazin-4-yl)-propyl-L-phosphonic acid ((R)-CPP) in the mPFC and also in the infralimbic cortex impaired accuracy and increased premature and perseverative responding, raising glutamate, dopamine, and GABA release in the dorsomedial striatum (Pozzi et al., 2011; Murphy et al., 2011; Agnoli et al., 2013). Similarly, in OCD patients, a dysregulation of glutamatergic signaling in the cortico-striatal circuitry has been suggested, with decreased concentrations of glutamate in the anterior cingulate cortex, accompanied by overactivity of the glutamate signaling in the striatum and orbitofrontal cortex (Pittenger et al., 2011; Ting and Feng, 2011; Milad and Rauch, 2012). Other authors proposed that the beneficial effect of MEM in OCD patients could be mediated by functional disconnection of the hippocampus with critical frontal regions (Vlček et al., 2018), by its effect on decreasing glutamate level in the hippocampus (Glodzik et al., 2009). Finally, we could hypothesize that according to these results, a possible explanation under the differences in compulsive HD rats selected by SIP might be an altered function of glutamatergic NMDA receptors that affect firing in cortical neurons in mPFC and affect glutamatergic, as well as dopaminergic and serotoninergic signal in the striatum.

## Conclusion

The exploration of other possible comorbid behaviors in compulsive HD rats selected by SIP indicated a relation with another form of compulsivity, measured by marble burying, and an increased vulnerability to cued fear behavior showed by an increased percentage of freezing time on FC compared to LD rats. No differences were found in the assessment of the depressive behavior on FST, nor in anxious behavior on EPM, replicating previous results from our laboratory (López-Grancha et al., 2008). The acute administration of glutamatergic drugs on SIP revealed that MEM and LAM dose-dependently and selectively decreased compulsive intake in HD rats, and did not affect LD on SIP. However, NAC did not affect compulsive drinking on SIP. These differences might be due to the specific action of the drugs on the presynaptic terminal. Further studies might disentangle the specific implication of the fear learning component and the dysregulation in glutamatergic neurotransmission, and its relation with the dopamine D_2/3_ and serotonergic 5-HT_2A_ receptors, in the mechanisms of vulnerability to compulsive behavior in HD rats on SIP.

## Ethics Statement

This study was carried out in accordance with the recommendations of “the Spanish Royal Decree 53/2013 on the protection of experimental animals, the European Community Directive (2010/63/EU) for animal experiments.” The protocol was approved by the University of Almería Animal Research Committee.

## Author Contributions

MM and PF designed research. ÁP-P, EM-G, SM and AM performed research. ÁP-P and EM-G analyzed data. ÁP-P and MM wrote the manuscript with the help of the other authors.

## Conflict of Interest Statement

The authors declare that the research was conducted in the absence of any commercial or financial relationships that could be construed as a potential conflict of interest.
